# A novel method for the percutaneous induction of myocardial infarction by occlusion of small coronary arteries in the rabbit

**DOI:** 10.1152/ajpheart.00657.2023

**Published:** 2024-01-05

**Authors:** Michael Freeman, Eline Huethorst, Erin Boland, Michael Dunne, Francis Burton, Chris Denning, Rachel Myles, Godfrey Smith

**Affiliations:** ^1^School of Cardiovascular and Metabolic Health, University of Glasgow, Glasgow, Scotland, United Kingdom; ^2^Biodiscovery Institute, University of Nottingham, Nottingham, United Kingdom

**Keywords:** cardiovascular disease, closed-chest model, intracoronary occlusion, myocardial infarction, percutaneous surgery

## Abstract

Arrhythmic sudden cardiac death (SCD) is an important cause of mortality following myocardial infarction (MI). The rabbit has similar cardiac electrophysiology to humans and is therefore an important small animal model to study post-MI arrhythmias. The established approach of surgical coronary ligation results in thoracic adhesions that impede epicardial electrophysiological studies. Adhesions are absent following a percutaneously induced MI, which is also associated with reduced surgical morbidity and so represents a clear refinement of the approach. Percutaneous procedures have previously been described in large rabbits (3.5–5.5 kg). Here, we describe a novel method of percutaneous MI induction in smaller rabbits (2.5–3.5 kg) that are readily available commercially. New Zealand White rabbits (*n* = 51 males, 3.1 ± 0.3 kg) were anesthetized using isoflurane (1.5–3%) and underwent either a percutaneous MI procedure involving microcatheter tip deployment (≤1.5 Fr, 5 mm), coronary ligation surgery, or a sham procedure. Electrocardiography (ECG) recordings were used to confirm ST-segment elevation indicating coronary occlusion. Blood samples (1 and 24 h) were taken for cardiac troponin I (cTnI) levels. Ejection fraction (EF) was measured at 6–8 wk. Rabbits were then euthanized (Euthatal) and hearts were processed for magnetic resonance imaging and histology. Mortality rates were similar in both groups. Scar volume, cTnI, and EF were similar between both MI groups and significantly different from their respective sham controls. Thus, percutaneous coronary occlusion by microcatheter tip deployment is feasible in rabbits (2.5–3.5 kg) and produces an MI with similar characteristics to surgical ligation with lower procedural trauma and without epicardial adhesions.

**NEW & NOTEWORTHY** Surgical coronary ligation is the standard technique to induce myocardial infarction (MI) in rabbits but is associated with procedural trauma and the generation of thoracic adhesions. Percutaneous coronary occlusion avoids these shortcomings and is established in pigs but has only been applicable to large rabbits because of a mismatch between the equipment used and target vessel size. Here, we describe a new scalable approach to percutaneous MI induction that is safe and effective in 2.5–3.5-kg rabbits.

## INTRODUCTION

Arrhythmic sudden cardiac death (SCD) remains a leading cause of mortality and occurs most frequently in patients with underlying coronary artery disease (CAD) (1, 2). Ventricular remodeling and scar formation, which occur after myocardial infarction (MI), disrupt normal cardiac electrophysiology and lower the threshold for ventricular arrhythmias (3). Preclinical models have an important role in the study of post-MI electrophysiology and can be used to develop novel diagnostic tools, risk stratification methods, and targeted therapies to improve clinical outcomes for patients with CAD (4–6).

Although large animal models may be theoretically preferable for preclinical studies, in practice their use is limited by cost, logistical, and ethical concerns (7). The rabbit is widely regarded as the optimal small animal model to study cardiac electrophysiology (8). In contrast to rats and mice, rabbits express the rapid (*I*_Kr_) and slow (*I*_Ks_) activating delayed rectifier potassium channels found in humans and arrhythmia dynamics are similar to those in humans (9). Furthermore, the rabbit heart has a comparable degree of coronary collateral circulation and therefore infarct structure (10). Development of left ventricular (LV) systolic dysfunction (LVSD), heart failure, and the incidence of SCD are also similar (11–14).

The standard approach to generating MI in animal models is surgical coronary ligation (4). This necessitates a thoracotomy, which in combination with the myocardial injury subjects the animal to significant procedural morbidity and mortality. In addition, division of the pericardium is necessary, resulting in epicardial adhesions that can affect scar remodeling (15, 16) and interfere with subsequent procedures or experimental studies (17).

Closed-chest models of MI, which use a percutaneous approach to coronary artery occlusion, are well-established in larger animals and recapitulate human MI more closely (18). Although there have been isolated reports on percutaneous induction of MI in rabbits (19–25), these techniques have not been widely adopted. The small size of rabbit vasculature makes the percutaneous approach a technical challenge. Multiple occlusion methods have been assessed and the most widely adopted method is the deployment of a coil (19, 22, 23, 25). This approach necessitates the use of microcatheters with an outer diameter of 0.83 mm (2.5 Fr) required to deliver the smallest commercially available embolization coils (0.018 in./0.46 mm). These microcatheters are not suitable for use in smaller rabbits (2.5–3.5 kg) where the coronary ostial diameter is 1.1 ± 0.3 mm (26). Notably, the largest study that deployed a coil percutaneously in the rabbit specified a weight range of 3.5–5.5 kg but did not report an average weight of animals in which the procedure was successful (*n* = 101) (22). For most researchers, routine use of such large rabbits would mean increased price, longer housing, the requirement for additional space, and adaptations to cage sizes (27) resulting in higher experimental costs.

Here, we provide a detailed description of a novel approach to percutaneous MI induction in 2.5–3.5-kg rabbits, including equipment selection and setup, perioperative safety, vascular access, angiography, and left coronary artery embolization. We demonstrate that catheter-tip deployment can be used in smaller coronary arteries. We also compare outcomes and infarct characteristics from percutaneous MI with surgical coronary ligation showing that our approach is not only feasible and safe but produces similar MI characteristics with lower procedural trauma and without epicardial adhesions.

Preliminary versions of these data were presented at a Physiological Society meeting by Dr. E. Huethorst (28).

## MATERIALS AND METHODS

### Ethics

The protocols used were approved by the UK Home Office and all work with animals conformed with the guidance on the operation of the Animals (Scientific Procedures) Act 1986. This work was done at the University of Glasgow under Project License PP5254544, Protocol 4. All procedures used male New Zealand White (NZW) rabbits (*n* = 51) (2.5–3.5 kg). The percutaneous MI protocol was developed under the direct supervision of the named veterinary surgeon (NVS).

### Percutaneous MI

Rabbits (*n* = 9) were premedicated, intubated, and ventilated using a small animal ventilator (Vetronic SAV04) with an oxygen condenser (delivering 81–100% O_2_ and targeting $\text{S}{\text{p}}_{{\text{O}}_{2}}$ > 95%). Core temperature was maintained above 37°C using a thermal mat and blanket. Following anesthetic induction, peripheral oximetry, capnography, electrocardiography (ECG), rectal temperature, and peripheral pulse pressure were monitored continuously. Following the introduction of a vascular sheath, blood pressure (BP) was also monitored invasively. Details on how physiological parameters were measured are provided in the standard protocol section. Physiological parameters were recorded using LabChart (AD Instruments).

The procedures were performed by a team including two sterile operators (*operators 1* and *2*) and a nonsterile anesthetist (*operator 3*). The target vessel was the left coronary artery that supplies the apex, which will be referred to as the “apical artery.” The exact anatomical classification of this vessel may vary between animals but is generally similar to the Ramus as described by Morrissey et al. (22). All procedures were performed in a sterile environment with the appropriate personal protective equipment. Fluoroscopic projections were acquired using a Philips BV Libra X-ray fluoroscopy system (C-Arm). Contrast dye (Iomeron 300, Iomeprol 61.24% wt/vol 300 mg iodine/mL, Bracco, UK) with heparinized saline (Hepsal; 100 U/mL) was in a 1:1 mixture for angiography, using the anterior-posterior projection. A full list of the surgical and angiographic equipment is given in Table 1. A supply of glycerol trinitrate (GTN) and adrenaline were available for use in case of hemodynamic collapse. A full list of the standard drugs used during this procedure is given in Table 2.

**Table 1.** Selection of wires and catheters used for the percutaneous procedure

Catheter Type	Size	Manufacturer	Catalog Number
Vascular sheath	4 Fr	Merit Medical	PHR4F7018SC
Guide wire	0.035 in.	Merit Medical	MSWSTDA35150
Angiographic catheter	4 Fr	Merit Medical	43035RIM
Floppy wire	0.007 in./0.008 in.	Baltsonic	Hybrid007D/Hybrid008D
Microcatheter	1.2 Fr/1.5 Fr	Baltsonic	Sonic1,2F25/Sonic1,5F25

Fr, French; in., inch.

**Table 2.** Summary of drugs used during the percutaneous and surgical MI procedures

Drug	Indication	Time of Administration	Dose	Administration Route
Heparinized saline (Hepsal)	Reduce thrombosis	Pre- and perisurgery	100 U/mL	Subcutaneous and intra-arterial
Xylocaine spray	Anesthetize oropharynx	Preintubation	10 mg/spray	Sublingual
Isoflurane	General anesthesia	During surgery	1.5–3%	Inhalation
Ketamine	Sedation	Presurgery	15 mg/kg	Subcutaneous
Medetomidine	Sedation	Presurgery	0.25 mg/kg	Subcutaneous
Atipamezole	Reverse medetomidine	Postintubation	1.25 mg/kg	Intramuscular
Carprofen	Analgesia	*Postoperative days 0, 1, 2*	4 mg/kg	Subcutaneous
Buprenorphine	Analgesia	*Postoperative days 0, 1, 2*	0.05 mg/kg	Subcutaneous
Metoclopramide	Promote gastric motility	Postoperative	0.1 mg/kg	Subcutaneous
Adrenaline	Increase inotropy	If hypotensive	0.1 mL/kg	Intra-arterial
Glycerol trinitrate	Treat coronary vasospasm	As required	2 μg/kg	Intracoronary
Quinidine	Anti-arrhythmic drug	As required	3–5 mg/kg	Intra-arterial or intravenous

The percutaneous MI sham procedure (*n* = 5) included all steps as shown in *Standard Protocol*, except *steps A7*, *A8*, *E5*, and *E6*. In brief, we instrumented the coronary artery with a wire, without blocking the vessel. After the wire was advanced into the coronary artery, the wire and angiographic catheter were removed, the wound was closed, and the animal recovered (as per steps in “*F*. *Wound closure and recovery*” of the protocol section).

### Surgical Coronary Ligation

The surgical ligation (*n* = 24) and corresponding sham procedures (*n* = 31) were performed as previously described (29–31). In brief, a left thoracotomy was performed through the fourth intercostal space under a general anesthetic. On exposing the LV by opening the pericardial sac, a major coronary artery, or apical vessel running on the basal/apical axis at the midline of the LV was identified and ligated approximately halfway between the ventricular groove and the cardiac apex with a suture around the artery and the 1–2 mm of surrounding myocardium. On successful ligation, an area distal to the ligature turned a dark blue color representing an ischemic area of ∼10% of the LV surface, in agreement with a previously published study (32). Sham-operated animals underwent thoracotomy and without coronary artery ligation. The protocol was developed over time while protocols were being established. Therefore, some data are missing from our data set, resulting in varying *n* numbers per experiment. The exact individual animal numbers are given for each experimental group.

### Verification of Coronary Occlusion

An ECG was acquired in lead I and II configuration, and successful coronary occlusion was determined by the development of characteristic ST-segment and/or T-wave changes.

### Postoperative Care

All animals received standard postoperative care, including analgesia (carprofen and buprenorphine) and metoclopramide (see Table 2). Following the procedure rabbits were transferred to enriched pens, where they received ad libitum food pellets, fresh fruit, vegetables, and water.

### Myocardial Infarction Biomarkers

One- and 24-h postocclusion, blood samples were taken from the marginal ear vein to measure cardiac troponin I levels (cTnI). Troponin measurements were performed at the Veterinary Biosciences Facility of the University of Glasgow. The protocol in brief was as follows: cTnI was measured using the IMMULITE 2000 Troponin I kit (Category No. L2KTI2, Siemens Healthcare) and quantified on the Immulite 2000Xpi system (Siemens Healthcare) (1-h cTnI: percutaneous MI, *n* = 8; surgical MI, *n* = 7; surgical sham, *n* = 3; and 24 h-cTnI, percutaneous MI, *n* = 8; surgical MI, *n* = 12; and surgical sham, *n* = 6). For percutaneous sham-operated animals, only the 24-h postoperative blood sample was taken (*n* = 5).

### Perioperative and Postprocedural Complications

Sustained ventricular arrhythmias were terminated with external defibrillation. An overview of abnormal ECG morphologies is shown in Fig. 6 and described in Table 3. A perioperative death was defined as occurring while the animal was under anesthesia. A postoperative death was defined as occurring between recovery from anesthesia and the planned euthanasia.

**Table 3.** List of adverse events and corresponding actions

Event	Class	Action
Ventricular ectopic beats	Mild	Watch ECG closely as this might be a precursor for arrhythmias. Check fluoroscopy for any damage of the aortic valve.
Cardiogenic shock/drop in blood pressure (>50%)	Moderate	Consider injecting IV fluids.Consider adrenaline bolus through the carotid sheath (Table 2).
Ventricular tachycardia	Moderate	Administer antiarrhythmic drugs (Table 2).
Ventricular fibrillation	Severe	Defibrillation. *Operator 1* operates the defibrillator, *operator 2* shocks the animal (30–50 J/shock), and *operator 3* assures removal of oxygen and detachment of sensitive electrical equipment (ECG adaptor) during the shock. Repeat up to three times if unsuccessful.
QRS broadening	Severe	Consider a bolus of glycerol trinitrate or adrenaline (Table 2). Euthanasia may be required.
Aortic rupture/tamponade	Lethal	Euthanasia
Cardiovascular collapse/asystolic cardiac arrest	Lethal	Euthanasia

ECG, echocardiogram; IV, intravenous.

### Endpoint Echocardiography, MRI, and Histology

Rabbits were maintained for 6–8 wk postoperation, until the myocardial scar was considered fully remodeled (17). At this point, echocardiography was performed to measure the LV size and function (percutaneous MI, *n* = 9; percutaneous sham, *n* = 4; surgical MI, *n* = 18; surgical sham, *n* = 19). Then, hearts went through a fixation process for ex vivo MRI and histology (percutaneous MI: *n* = 5; percutaneous sham: *n* = 2; surgical MI: *n* = 14; or surgical sham: *n* = 4). In brief, rabbits were administered terminal anesthesia through the marginal ear vein using sodium pentobarbitone (Merial, 200 mg/kg) mixed with 1,000 IU heparin. Beating hearts were rapidly excised and immediately placed in ice-cold Tyrode’s solution to stop contraction. The aorta was then cannulated directly onto a continuous perfusion system, secured, and the heart perfused for 10 min with ice-cold Tyrode’s solution, containing (in mM) 135 Na^+^, 5.0 K^+^, 1.9 Ca^2+^, 1.0 Mg^2+^, 101.8 Cl^−^, 1.0 $SO_{4}^{2-}$, 0.7 ${\text{HPO}}_{4}^{2-}$, 20 ${\text{HCO}}_{3}^{-}$, 15 acetate (CH_3_COO^−^), and 25 glucose, followed by 15 min with 5% paraformaldehyde solution. Fixed hearts were transferred into a Fomblin-filled syringe and scanned with a 7-T Bruker Biospin PharmaScan70/16 MRI, 110-A gradient amplifiers. Gradient echo (FLASH, T1) and turbo spin-echo (Rapid Acquisition with Refocused Echoes, RARE, T2) based sequences were optimized for structural imaging of the heart (repetition time/time to echo/flip angle: 15 ms/4.804 ms/30/matrix: 288 × 288 × 400/field of view: 36 mm × 36 mm × 50 mm, resolution: 125 µm^3^). The scan time was 12 h. Fixed hearts were then embedded in paraffin for Masson’s trichrome staining to visualize scar and surviving myocardium. All images were processed and analyzed using ImageJ and 3D Slicer. Vessel diameters were calculated in two perpendicular directions in the axial plane. The occlusion site was determined as the coronary vessel diameter immediately proximal to either the surgical ligation site or the microcatheter tip location. MRI scans were segmented using a semiautomated algorithm available in 3D Slicer (LV, scar, and other myocardium). These data were used to estimate LV and scar volumes (33). Coregistered histological sections were used to determine the signal intensity ranges for normal and abnormal myocardium and visually assess the accuracy of segmentations.

### Standard Protocol

#### A. Percutaneous kit preparation.

**Figure 1.**
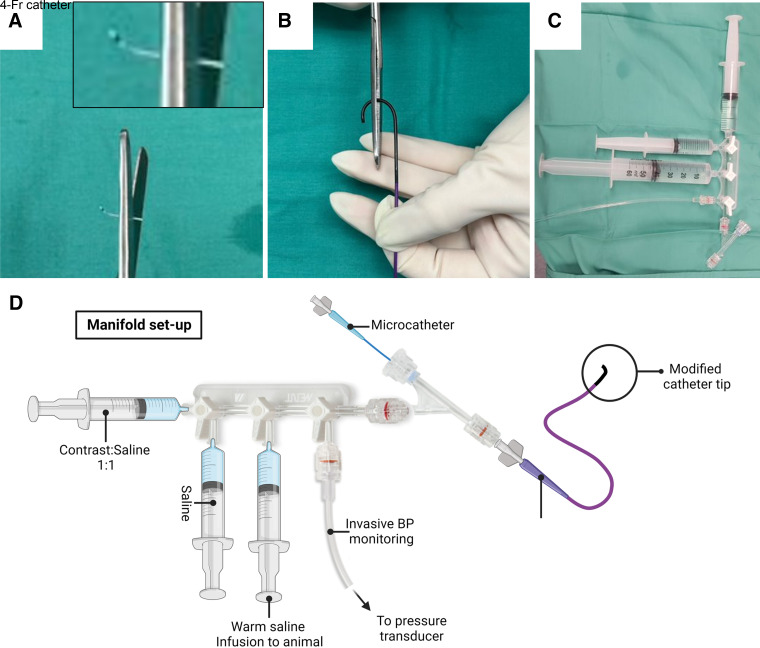
Preparing the manifold and monitoring setup. *A*: modification of the microcatheter tip to a 5 mm length. *B*: modification of the 4-Fr angiographic catheter for use in the rabbit. *C* and *D*: typical manifold setup as a photograph (*C*) and diagram (*D*). Microcatheter and angiographic catheters are shown in *D* for reference but would not be attached until a later point in the procedure. BP, blood pressure.

1.  *Operator 1*: don mask/sterile scrub/gown/gloves.
2.  *Operator 1*: prepare a sterile field on the operating table and place two containers onto the field.
3.  *Operator 3*: pour warmed (39°C) heparinized saline (Hepsal, Table 2) into the two containers; one for clean equipment, one for used equipment.
4.  *Operator 3*: check equipment and verify with *operator 1* before opening for *operator 1*.
5.  *Operator 1*: take on sterile equipment and prime with warm Hepsal, remove bubbles, and place in the Hepsal bath ready for use.
6.  *Operator 1*: cut the tip of the angiography catheter (4 Fr) to ensure a 90° angle and that the distal end is approximately equivalent to the aortic diameter (4–5 mm) (Fig. 1*B*).
7.  *Operator 1*: cut the microcatheter’s distal tip (1.2/1.5 Fr) including the radiopaque marker. Then trim this further until this separated tip is 5 mm in length and includes the radiopaque marker.
8.  *Operator 1*: load both the microcatheter and tip onto the micro-guidewire (0.007 in./0.008 in.), where the radiopaque marker is positioned proximally because this will be the indication of the proximal portion of the infarct. Leave ∼10–15 cm of wire distal to the tip to ensure the tip does not deploy inadvertently.
9.  *Operator 1*: setup the manifold as indicated in Fig. 1. Attach three 20-mL Luer-lock syringes containing *1*) Hepsal-contrast agent mix, *2*) and *3*) Hepsal, pressure transducer, and the Y-piece hemostatic valve. Prime the full system, including all tubing, with Hepsal.

#### B. Anesthetic induction and surgical preparation.

**Figure 2.**
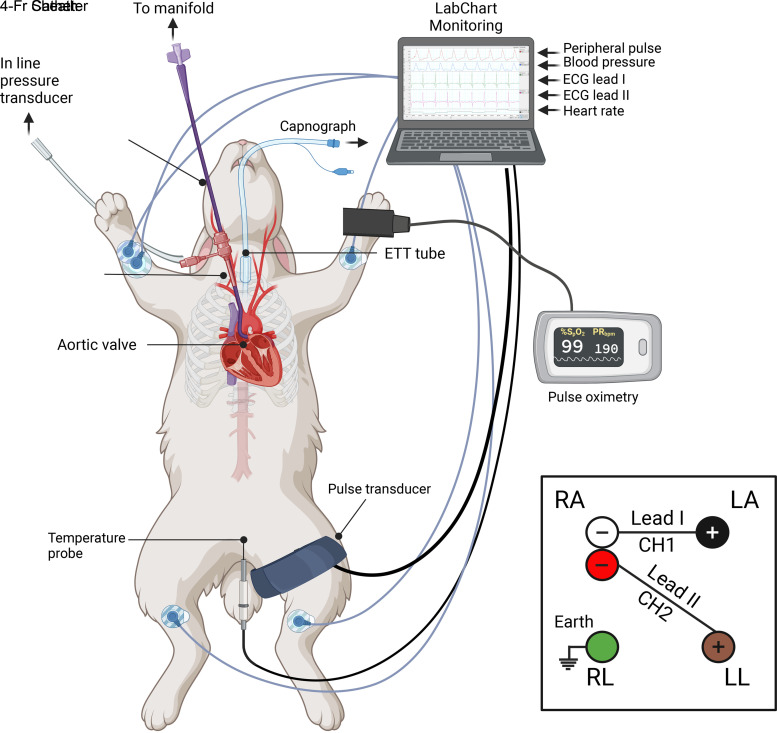
Diagram illustrating all animal monitoring during the procedure. *Inset*: overview of electrocardiography (ECG) lead I and II configuration. ETT, endotracheal tube; RA, right atrium; LA, left atrium; RV, right ventricle; LV, left ventricle.

1.  *Operator 2*: administer ketamine and medetomidine subcutaneously to sedate the animal (Table 2).
2.  *Operators 2* and *3*: after 10 min use clippers to remove all hair from the anterior chest, neck, ears, and front and hind limbs of the animal.
3.  *Operator 2*: intubate the animal using a laryngoscope and endotracheal tube (ETT). Confirm successful intubation using *1*) auscultation of the breath sounds, *2*) capnography, and *3*) pulse oximetry.
4.  *Operator 3*: ventilate the animal using 3% isoflurane. Adjust the expiratory time and pressure to optimize end-tidal CO_2_ (30–40 mmHg) and respiration rate (30–40 breaths/min) as shown on the capnograph. Place the pulse oximeter on the front paw of the rabbit to measure peripheral O_2_ saturation (95–100%).
5.  *Operators 2* and 3: with the rabbit in a supine position, secure the ETT. Use sandbags and a thermal blanket to stabilize and insulate the rabbit, respectively.
6.  *Operator 3*: once stable anesthesia has been achieved, administer the intramuscular atipamezole (Table 2) to reverse the effects of medetomidine, and then gradually reduce the isoflurane to 1.5–2.5%. Isoflurane concentration is monitored with capnography during the procedure.
7.  *Operator 2*: secure a venous cannula in the left marginal ear vein and use this to administer 10 mL Hepsal with 200–300 U of heparin.
8.  *Operator 3*: position the ECG electrodes (lead I and II), the pulse pressure monitor (femoral artery), and the anal temperature probe as shown in Fig. 2.
9.  *Operator 2*: prepare the surgical site with an iodine scrub and cover the remaining surgical field with a sterile keyhole drape.
10.  *Operator 2*: scrub in and finish the sterile surgical field.
11.  *Operator 3*: liaise with *operator 1* whether help is needed, then take place behind the anesthesia equipment. From now on, study the physiological parameters and adjust the anesthesia accordingly. Make notes of important events, such as the introduction of the sheath or catheters. Assist *operator 1* and *operator 2* with nonsterile activities where needed, e.g., positioning the C-arm.

#### C. Vascular access.

**Figure 3.**
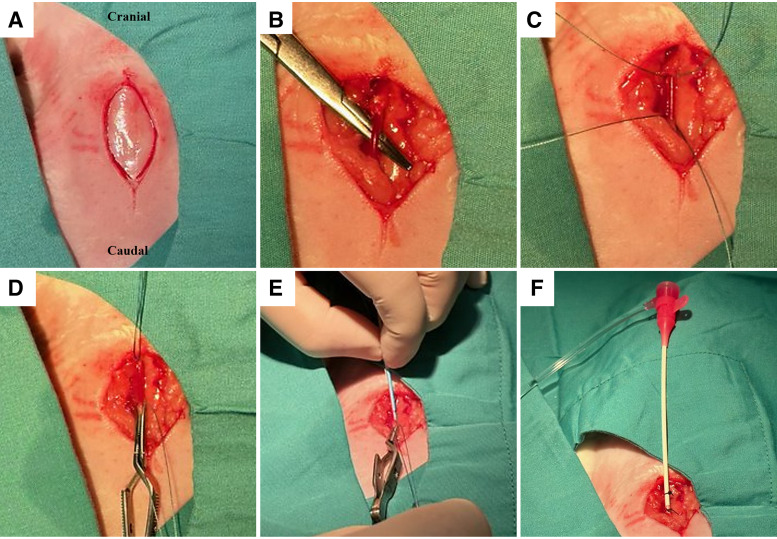
Vascular access to the carotid artery. *A*: 3*–*4 cm midline incision. *B*: carotid artery is isolated using blunt dissection. *C*: two sutures are passed under the exposed isolated carotid artery. *D*: knot is tied in the suture at the cranial end, and a bulldog clamp used to occlude the caudal end. *E*: vascular sheath is introduced through a small incision in the isolated carotid; once the bulldog clamp is released, the second operator is ready to stop backflow of blood. *F*: sheath is secured in position with two sutures.

Note: An alternative to dissection of the carotid artery is to use a Seldinger approach, i.e., introducing a guide wire through a needle in the vessel, removing the needle and passing a sheath over it. When introducing the vascular sheath, care is required to avoid damage to the carotid artery. Vessel closure without direct visualization is also technically challenging.

1.  *Operator 1*: place the introducer within the vascular sheath and prime with Hepsal.
2.  *Operator 2*: using a scalpel, make a 4-cm midline incision from the tracheal notch (Fig. 3*A*).
3.  *Operator 2*: use blunt dissection to expose 2–3 cm of the internal carotid artery. It is important to take care to locate and separate the vagus nerve that is anterior to the carotid artery (Fig. 3*B*).
4.  *Operator 2*: tie a suture (3-0 vicryl) to occlude the cranial end of the exposed carotid artery. Place a second untied suture under the exposed carotid at the caudal end. This portion of the vessel should also be occluded temporarily using a bulldog clamp (Fig. 3, *C* and *D*).
5.  *Operator 2*: make a small linear cut in the middle of the occluded carotid using fine scissors.
6.  *Operator 2*: using fine bracket-holding tweezers to hold the artery open, insert the sheath and introducer into the lumen of the carotid (Fig. 3*E*). *Operator 1* assists.
7.  *Operator 2*: carefully remove the bulldog clamp and advance the sheath further into the vessel. Confirm backflow and then cover the back of the introducer to avoid bleeding.
8.  *Operator 2*: after 1 cm of the sheath is inside the carotid artery carefully remove the introducer, while simultaneously advancing the sheath a further 1–2 cm to a secure position (Fig. 3*F*).
9.  *Operator 1*: flush the introducer in one Hepsal bath to avoid cloth formation inside the introducer. Do this with all equipment returned from the surgical site.
10.  *Operator 2*: suture the sheath to the skin. It is imperative that the sheath is well secured for the remainder of the procedure. Keep the wound covered with a wet swab for the remainder of the procedure (Fig. 3*F*).
11.  *Operator 2*: with the use of a 10-mL syringe containing Hepsal, aspirate blood from the side port of the sheath, isolate any bubbles in the syringe and flush the sheath with Hepsal.
12.  *Operator 1*: measure arterial BP from the carotid sheath.

#### D. Angiography.

**Figure 4.**
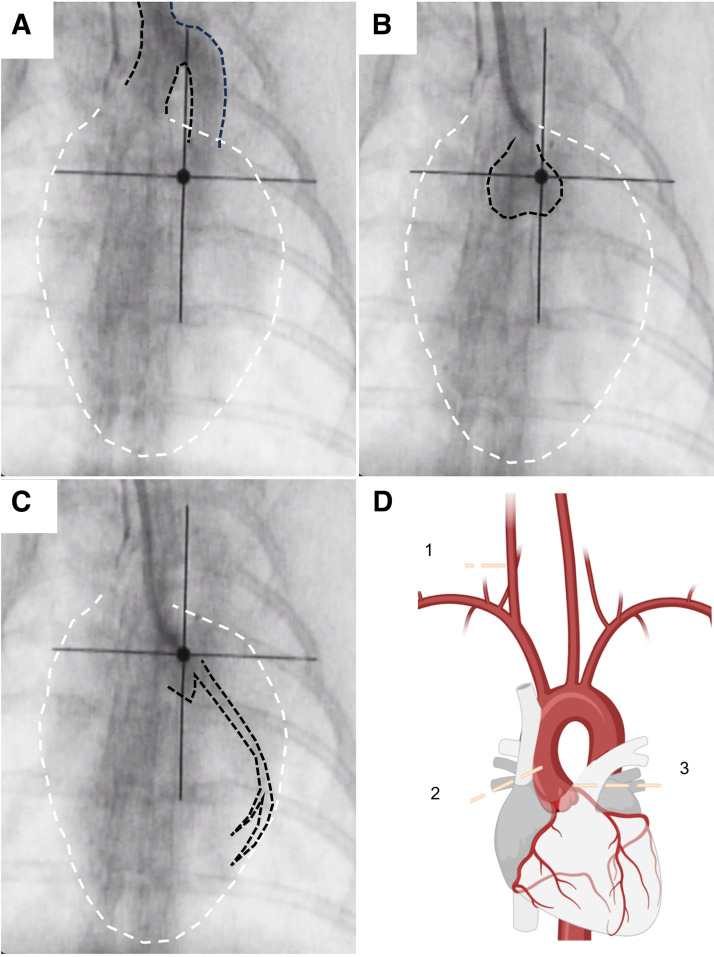
Fluoroscopic images made during the percutaneous procedure directly after contrast dye injection. *A*: contrast is injected into the carotid via the sheath giving an outline of the aortic arch. *B*: contrast is injected from the 4-Fr angiographic catheter while positioned in the ascending aorta to outline the aortic valves. *C*: contrast is injected directly into the left ostium through the 4-Fr angiographic catheter to show the course of the apical artery and major branches. Dashed black lines in *A–C* indicate the aortic arch, aortic valve, and left ostium/coronary artery, respectively. Dashed white lines indicate the outline of the rabbit heart. *D*: diagram showing an overview of the various angiographic steps along with the vascular anatomy of the heart. Numbers indicate locations where contrast dye is injected. *Injection sites 1*, *2*, and *3* correspond to *images A*, *B*, and *C*, respectively.

Note: Keep the three-way tap on the manifold, proximal to the rabbit, closed unless recording BP or performing injections. This will prevent further bleed back into the manifold and keep the catheter clear of blood.

1.  *Operator 3*: position the C-arm over the rabbit thorax using an anterior-posterior projection, assuring the field of view includes the cardiac contour and supracardiac vasculature within the thorax (Fig. 4).
2.  *Operator 1*: while recording, rapidly inject 2–3 mL of the Hepsal-contrast mix into the side port of the vascular sheath, followed by 3 mL of a Hepsal flush. This angiogram will delineate the vascular structures connecting the carotid artery, aortic arch, and ascending aorta (Fig. 4*A*).
3.  *Operator 1*: using the introducer, place a guidewire (0.035 in.) into the sheath and use the roadmap from *step D2* to advance approximately one rib space into the ascending aorta to avoid crossing the aortic valve.
4.  *Operator 2*: hold the guidewire to fix it in place.
5.  *Operator 1*: prime the angiographic catheter with Hepsal and advance it over the fixed guidewire. Fluoroscopy can be used to confirm the position of the guide wire does not change.
6.  *Operators 1* and *2*: once the angiographic catheter is positioned in the ascending aorta, hold it in place and remove the guidewire under fluoroscopic guidance. Then, rotate the angiographic catheter so that the end is pointing to the left side of the ascending aorta/ostium.
7.  *Operator 1*: attach the primed manifold to the back of the catheter. Aspirate, isolate any bubbles in the syringe, and flush Hepsal through the manifold and catheter.
8.  *Operator 1*: take the pressure transducer from the side port of the sheath and connect it to the manifold system to allow for direct measurement of aortic BP.

1.  *Operator 1*: while recording fluoroscopic images, rapidly (1–2 s) inject a (1 mL) bolus of the Hepsal-contrast mix through the angiographic catheter. This image should delineate the ascending aorta, including the position of the aortic valve (Fig. 4*B*).
2.  *Operator 1*: move the angiographic catheter into position with the catheter tip coaxial to the left coronary ostium. Use further small (0.5 mL) injections of Hepsal-contrast mix to optimize this position. The optimal position for angiography is indicated by *1*) catheter tip motion, i.e., a jump as the catheter engages the coronary ostium and *2*) isolated contrast seen in the left apical artery (Fig. 4*C*). Flush with Hepsal afterward.
3.  *Operator 1*: while recording fluoroscopic images, inject a larger volume of contrast (1–2 mL) rapidly to identify the anatomical course of the left coronary system, including the number of major left-sided vessels, branching and size. Flush with Hepsal after.
4.  *Operator 1*: retract the angiographic catheter slightly away from the coronary ostium. Be careful not to pull this as far as the aortic arch as this will necessitate the use of the guide wire (0.035 in.) to reposition. It is important to minimize the time the angiographic catheter is covering the coronary ostium to minimize complications.

Note: Example fluoroscopic videos of these procedural steps can be found in the Supplemental Video File.

#### E. Occlusion of the coronary artery.

**Figure 5.**
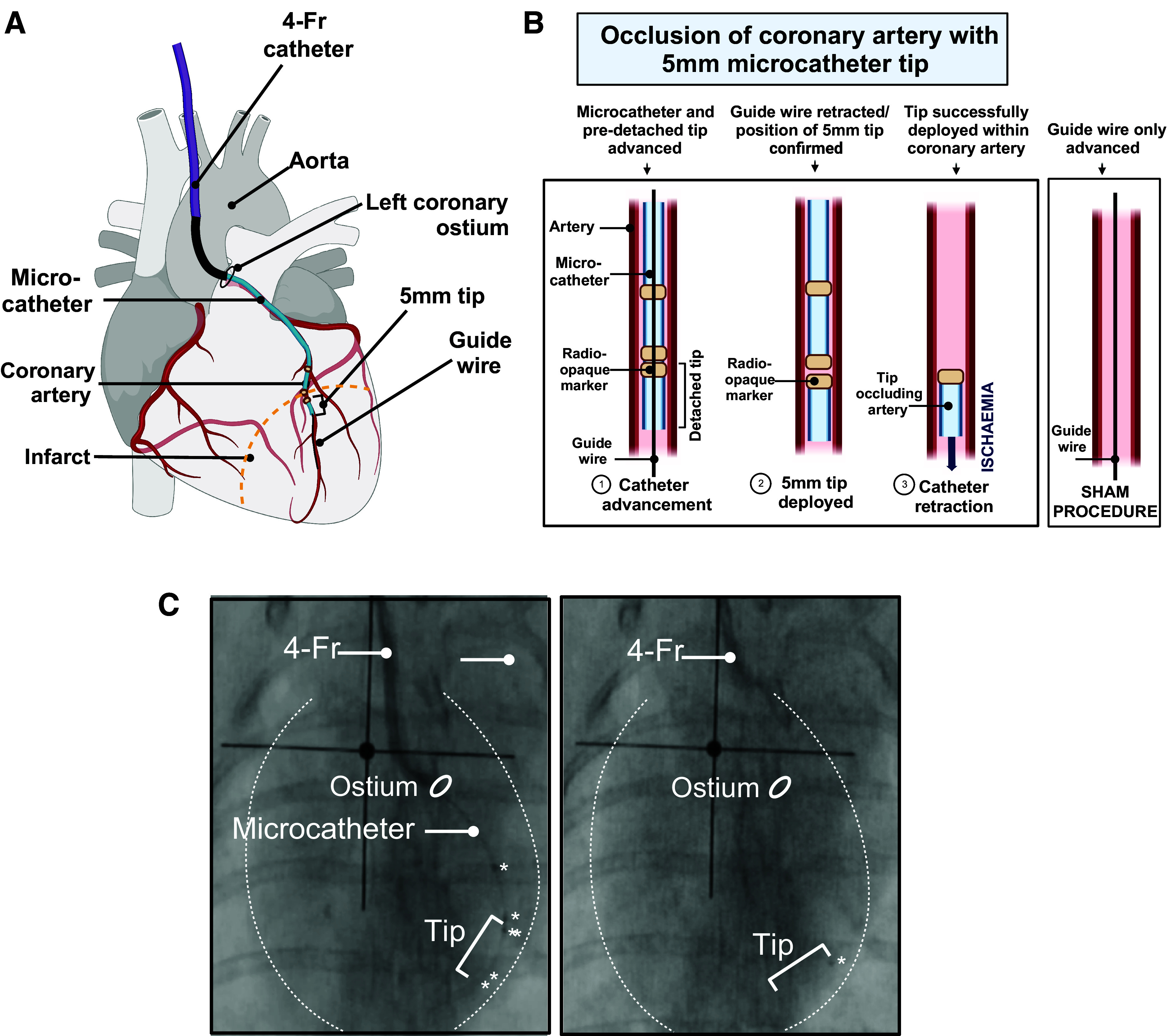
Occlusion of a coronary artery with a microcatheter tip. *A*: diagram showing the microcatheter being advanced into the apical artery through the 4-Fr angiographic catheter. *B*: detailed diagram showing tip deployment within the apical artery. *C*: fluoroscopic projections indicating the stages in tip deployment: during (*left*) and after deployment (*right*). Radiopaque markers on the microcatheter and tip are indicated with an asterisk, and the dotted white line represents the cardiac contour.

Note: Be careful to minimize blood loss when the valve is opened and closed.

1.  *Operators 1*, *2*, and *3*: A schematic overview of the tip-deployment procedure is shown in Fig. 5, *A* and *B*. Determine the target occlusion site using the angiogram made in *step D11*. In general, a moderate-sized infarct will be generated when placing the block two-thirds along the length of the apical artery that runs along the lateral border of the cardiac silhouette in the anterior-posterior projection at ∼2 rib spaces below the aortic valve (22). An example is shown in Fig. 5*C*, *right*.
2.  *Operator 1*: advance the microcatheter, tip and wire as a unit (see *step A8*) into the side port of the hemostatic valve using an introducer, while *operator 2* assures the position of the 4-Fr catheter.

1.  *Operators 1* and *2*: use fluoroscopy to determine when the micro-guidewire is near the distal end of the angiographic catheter. At this point the angiographic catheter should be manipulated back into the position where an angiogram was performed, i.e., into or near the ostium.

Note: it is not possible to perform angiography with the microcatheter in situ, so the key sign to visualize is the “jump” in the catheter as it falls into the ostium.

1.  *Operator 1*: advance the micro-guidewire into distal the apical artery. This should follow the same trajectory as the angiogram.

**Figure 6.**
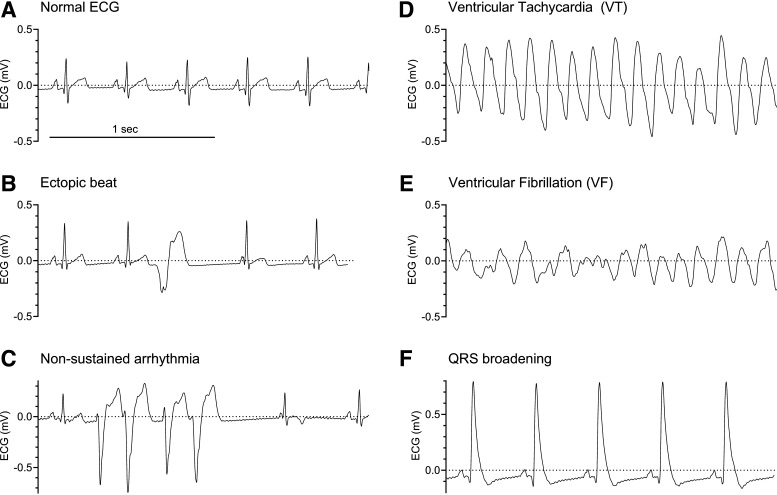
Example electrocardiography (ECG) traces. *A–F*: normal sinus rhythm (*A*), sinus rhythm with a single ectopic beat (*B*), 4-beat burst of ventricular ectopy during sinus rhythm (*C*), polymorphic ventricular tachycardia (VT; *D*), ventricular fibrillation (VF; *E*), and sinus rhythm with QRS broadening (*F*). Treatment of arrhythmias and QRS broadening is outlined in Table 3.

Note: Example fluoroscopic videos of these procedural steps can be found in the Supplemental Video File.

Note: if the micro-guidewire does not enter the coronary artery, the angiographic catheter may need to be rotated or moved to adjust its position. It is important that the wire does not instead pass into the LV. Monitoring ECG for ventricular ectopy (Fig. 6*B*) may be indicative that the aortic valve has been crossed. The micro-guidewire may be easily damaged and if it is not easily advanced into the coronary artery, it should be withdrawn to check its integrity. In this case, *steps D12*–*E4* should be repeated, minimizing the time the angiographic catheter is over the coronary ostium. If the wire tracks into a side branch, it should be withdrawn and repositioned. It may also be necessary to perform an angiogram again (*steps D10* and *D11*)

1.  *Operators 1* and *2*: once the wire is in the distal apical artery, *operator 2* fixes it and *operator 1* advances the microcatheter and tip over the wire under fluoroscopic guidance to ensure the wire does not move forward, risking distal vessel perforation/dissection.
2.  *Operator 1*: the radiopaque tip should be pushed using the microcatheter until it reaches the desired location determined during *step E1*.
3.  *Operator 2*: remove the micro-guidewire. It is then safe to remove the microcatheter, followed by the angiographic catheter.
4.  *Operator 3*: pay attention to any ECG changes, including ST-elevation, ST-depression, or T-wave inversion. These can be noted immediately but may take several minutes to develop.
5.  *Operator 1*: perform a final fluoroscopic recording to document the final position of the tip.
6.  *Operator 1*: unscrub to assist during potential adverse effects as described in Table 3 and shown in Fig. 6. *Operator 2* stays sterile.

#### F. Wound closure and recovery.

1.  *Operator 1*: switch the pressure transducer back onto the vascular sheath to monitor BP for a minimum of 25 min after tip deployment. In case of arrhythmias or other adverse effects, refer to *G*. *Potential adverse effects*.
2.  *Operator 2*: begin wound closure when the BP and peripheral pulse pressure are stable for 25 min postocclusion with no ventricular arrhythmias. The animal should remain anesthetized and monitored for arrhythmias for a minimum of 1 h.
3.  *Operator 2*: place a suture underneath the caudal end of the exposed carotid artery and tie a loose overhand knot. Remove any sutures that were used to fix the sheath in position.
4.  *Operators 1* and *2*: *operator 1* carefully pulls back to remove the sheath and BP sensor, while *operator 2* pulls the overhand knot tight to prevent blood loss and tie off the carotid artery. A bulldog clamp can also be used temporarily if necessary.
5.  *Operator 2*: close the various layers of the wound. Interrupted sutures can be used to close deeper muscle layers and continuous subcuticular sutures to close superficial skin layers.
6.  *Operator 2*: once the wound is closed, position the animal in the left lateral position. If stable for at least 45 min postocclusion, begin to progressively reduce the isoflurane and progress toward extubation and full recovery.
7.  *Operator 2*: remove all ECG leads, the peripheral pulse pressure, and the temperature probe. The pulse oximeter can be used to monitor heart rate (HR) and oxygen saturation during the final minutes of the recovery.

#### G. Potential adverse effects.

Arrhythmias are most likely to develop 15–25 min after occlusion but may occur up to 1-h postocclusion (19, 25, 34). Table 3 summarizes the adverse events and corresponding management. Figure 6 shows examples of the various abnormal ECG changes described in Table 3.

## RESULTS

### Coronary Artery Diameter Measurements for Selection of Angiographic Equipment

**Figure 7.**
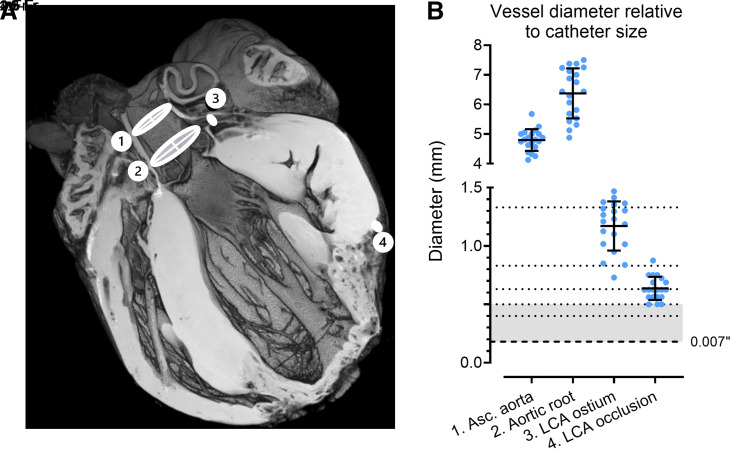
Vessel diameter relative to various catheter sizes. *A*: example T1w MRI image of a heart that underwent percutaneous occlusion. *1*) Axial cross-section includes ascending aorta, *2*) aortic root, *3*) left coronary artery (LCA) ostium, and *4*) LCA occlusion site in the apical artery. *B*: quantification of vessel diameters of hearts (*n =* 20) that were either used for ligation or percutaneous myocardial infarction (MI) procedures. Dotted lines represent relative sizes of angiographic- and microcatheters, and dashed line indicates the micro-guidewire. Gray zone represents the range of catheters and wires used in this study. Vessel diameter was measured just proximal to No. 4. Data are shown as means ± SD.

To determine what angiographic equipment would be suitable for use in rabbit vasculature, a series of ex vivo MRI scans were performed. A total of 20 T1-weighted MRI scans of hearts following an MI procedure were used to measure the diameter of the ascending aorta, the aortic root, the left coronary ostium, and the location just above the occlusion site, as shown in Fig. 7*A*. The measurements obtained from these scans are presented in Fig. 7*B* along the sizes of various percutaneous equipment and were as follows: ascending aorta diameter 4.80 ± 0.37 mm, aortic root diameter 6.38 ± 0.85 mm, coronary ostium diameter 1.17 ± 0.21 mm, and occlusion site diameter 0.64 ± 0.10 mm. These aortic dimensions were used to determine the angiographic catheter size. Considering the smallest occlusion site diameter of 0.50 mm, it was determined that catheters of 1.5 F or smaller would be most appropriate for use in rabbit coronaries.

### Operative Success Rate

A total of 14 rabbits underwent a percutaneous procedure, with five receiving sham interventions (3.3 ± 0.1 kg) and nine receiving catheter tip embolization (3.2 ± 0.3 kg). In the percutaneous MI group, one postoperative death occurred at 8 wk. According to the postmortem investigation, the cause of death was most likely related to the MI. In comparison, a total of 24 animals underwent surgical ligation (3.1 ± 0.3 kg) and another 19 animals received the corresponding sham surgery (3.1 ± 0.3 kg). In the surgical MI group, 8% of animals died postoperatively (2/24; *P* > 0.99 vs. percutaneous MI). An overview of weight ranges and mortality rates is shown in Table 4.

**Table 4.** Average values of weights, mortality rates, and indicators of MI of rabbits undergoing MI or sham procedures

	Percutaneous					Surgical					*P* Value	
	MI		Sham		*P* value	MI		Sham		*P* value	MI	Sham
		*n*		*n*			*n*		*n*			
Total *n*	9		5			24		19				
Weight, kg	3.2 ± 0.3		3.3 ± 0.1		0.97	3.1 ± 0.3		3.1 ± 0.3		0.88	0.74	0.83
Range (min–max), kg	2.9–3.6		3.1–3.4		NA	2.5–3.7		2.5–3.5		NA	NA	NA
*Mortality rates*												
Postoperative death, *n* (%)	1/9 (11)		0/5 (0)		>0.99	2/24 (8)		1/19 (5)		>0.99	>0.99	>0.99
*Indicators of MI (subsets of animals)*												
Range cTnI, ng/mL												
1 h*	<0.225	8	<0.2	2	NA	1.13–9.03	7	<1.52	3	**0.0083**	**0.0002**	NA
24 h§	>46.8	8	<0.890	5	**0.0005**	>37.1	12	0.54–1.6	5	**0.0002**	>0.99	>0.99
End-point EF, %	49 ± 8	9	66 ± 4	4	**0.0008**	48 ± 7	18	64 ± 7	19	**<0.0001**	0.94	0.89
Scar volume, %LV	8.5 ± 6.2	5	0.0 ± 0.0	2	NA	8.4 ± 3.7	18	0.0 ± 0.0	4	NA	>0.99	NA

Values are means ± SD, ranges, or *n* (%). For the weights, ejection fraction (EF), and scar size, data were analyzed using a one-way ANOVA with a Tukey post hoc test. For cardiac troponin I (cTnI) and the overall mortality rate, data were categorized and analyzed using a two-sided Fisher’s exact test. LV, left ventricle; MI, myocardial infarction; min–max, minimum to maximum.

* Data were categorized with a 1 ng/mL cutoff value; §data were categorized with a 10 ng/mL cutoff value. *P* < 0.05, significant difference (shown in boldface).

### Confirmation of MI following Catheter Tip Deployment

**Figure 8.**
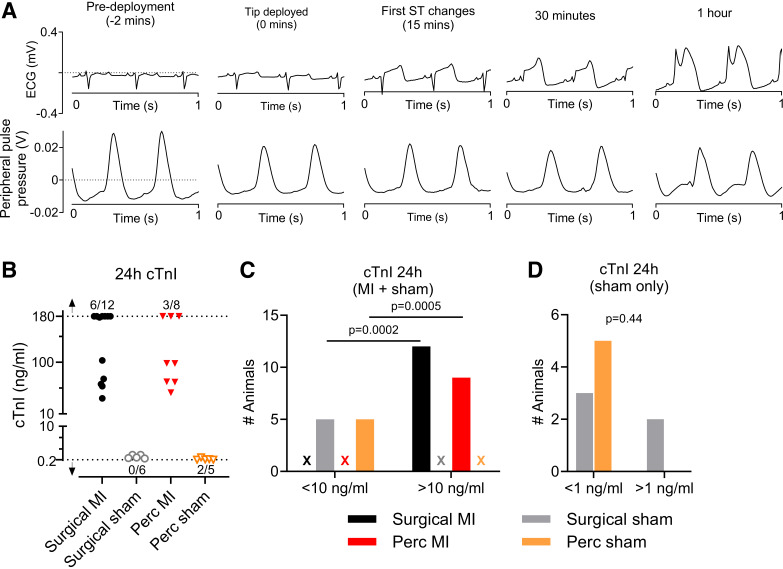
Infarct characterization and comparison of surgical myocardial infarction (MI)/sham and percutaneous MI/sham. *A*: progression of ST-segment changes after tip deployment with associated peripheral pulse pressure measurement. *B*: 24-h troponin levels for MI and sham procedures. Troponin analysis detects cTnI within a range of 0.2–180 ng/mL. Arrows indicate range of values below or above the detection range. Ratios next to the arrow indicate number of values out of total number of data points that were out of range. Categorical analysis was done with a cutoff value of 10 ng/mL (*C*) or 1 ng/mL (*D*). *C*: two-sided Fisher’s exact test showed surgical MI vs. sham, *P* = 0.0002; percutaneous MI vs. sham: *P* = 0.0005, both MI groups compared, *P* > 0.99; both sham groups compared, *P* > 0.99. Crosses indicate absence of values in that category. *D*: two-sided Fisher’s exact test for both sham groups compared with a [cardiac troponin I, cTnI] cutoff of 1 ng/mL resulted in a *P* value of 0.44. Values in *D* are from sham groups only.

After deploying the catheter tip, the presence of MI was confirmed by observing ST-segment elevation in the ECG, as shown in a sample trace in Fig. 8*A*. Importantly, peripheral pulse pressure was stable over the course of the procedure (Fig. 8*A*, *bottom*). Twenty-four-hour cTnI levels are shown in Fig. 7*B*. However, because the troponin assay has a detectable range with lower and upper limits between 0.02 and 180 ng/mL, respectively, it was decided to categorize the data using a [cTnI] cutoff value of 10 ng/mL to perform statistical assessment (Fig. 8*C*). Here, all MI groups showed cTnI levels above 10 ng/mL, whereas the levels in the sham group were below this cutoff level (surgical vs. sham: *P* = 0.0002; percutaneous vs. sham: *P* = 0.0005). No significant differences were seen when comparing both sham or both MI groups (*P* > 0.99). To investigate the surgical damage in more detail, both sham groups were additionally categorized with a [cTnI] cutoff value of 1 ng/mL. All values of the percutaneous sham group were below 1 ng/mL, whereas only 60% of the values for the surgical sham group fell below 1 ng/mL (*P* = 0.44). These data indicate that the percutaneous MI surgery induces myocardial damage comparable with that of the surgical MI procedure and that the surgical procedure, but not the percutaneous procedure, results in nonischemia-related myocardial damage.

### Effects of Percutaneous Intervention at 6–8 wk

**Figure 9.**
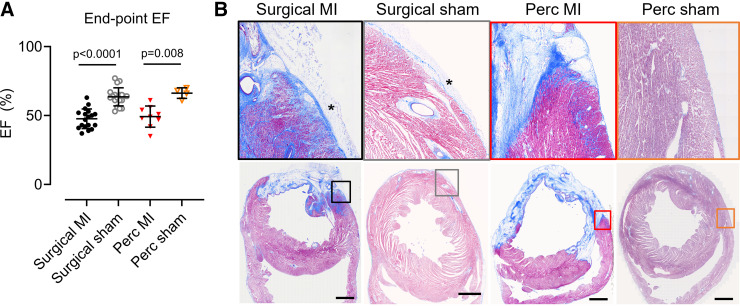
Comparison of the myocardial infarcted (MI) hearts 6–8 wk postocclusion. *A*: ejection fraction (EF). Bars show means ± SD. One-way ANOVA with Tukey’s post hoc test. *B*: Masson’s trichrome staining of rabbit hearts subjected to either a surgical sham, surgical MI, percutaneous sham, or percutaneous MI procedure. *Top*: enlarged sections (3 × 3 mm) of images at *bottom*. Scale bar indicates 3 mm. *Adhesions.

After 6–8 wk LV function was assessed by echocardiography (Fig. 9*A*). Percutaneous MI procedures resulted in a significant reduction in EF (49 ± 8 vs. 66 ± 4%, *P* = 0.0008). Following surgical MI, EF was also reduced (48 ± 7 vs. 64 ± 7%, *P* < 0.0001). There was no difference between EF in percutaneous or surgical MI groups (*P* > 0.99).

Histology indicated that both the percutaneous and surgical MI procedures resulted in a transmural scar (Fig. 9*B*). No scar formation was seen in either sham group. Hearts from animals undergoing thoracotomy displayed adhesions on the epicardial surface, which was absent in both the percutaneous MI and sham groups (Fig. 9*B*, indicated with asterisk).

**Figure 10.**
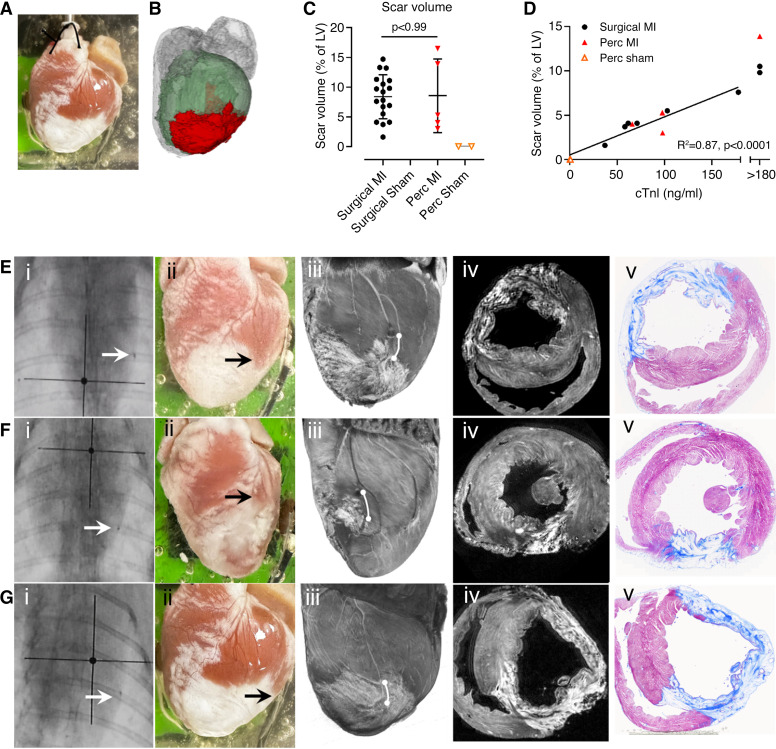
Quantification of scar size. *A*: macroscopic image of a heart. *B*: segmented MRI render of the same heart as in *image A*; green indicates left ventricle (LV), and red, the scar area. Gray areas are the atria and right ventricle. *C*: scar volume quantification as percentage of the LV. Statistics were done using a one-way ANOVA with Tukey’s post hoc test. Bars show means ± SD. *D*: linear regression of scar volume with 24-h troponin values and scar volumes of hearts where the cardiac troponin I (cTnI) levels were >180 ng/mL. *E–G*: typical infarct patterns in three different hearts subjected to percutaneous tip deployment. Each row represents a different heart: *i*) anterior-posterior fluoroscopic projection, arrow indicating the radiopaque tip marker; *ii*) gross appearance of scar on the epicardium; *iii*) T2w MRI volume render showing the lateral free wall of the LV (tip position is indicated by white line. It is possible to see where the scar formed in relation to the blocked coronary); *iv*) axial slice from T2w MRI; and *v*) corresponding histology with Masson’s trichrome stain.

Scar volumes were quantified using three-dimensional (3-D) MRI scans. Figure 10*A* shows an image of a heart following percutaneous MI, with the corresponding MRI image used for analysis in Fig. 10*B*. There was no significant difference between the surgical MI and the percutaneous MI (8.4 ± 3.7 vs. 8.5 ± 6.2%; *P* > 0.99), and no scar was detected in either sham group (Fig. 10*C*). Twenty-four-hour cTnI and scar volumes were positively correlated as shown in Fig. 10*D.* Figure 10, *E*–*G*, shows three examples of hearts that underwent the percutaneous MI procedure where the tip was deployed at a similar location visually, as shown on the fluoroscopic images. However, all three hearts resulted in a variable scar morphology as shown by macroscopic images, 3-D MRI, and histology.

### Myocardial Injury and Epicardial Adhesions

**Figure 11.**
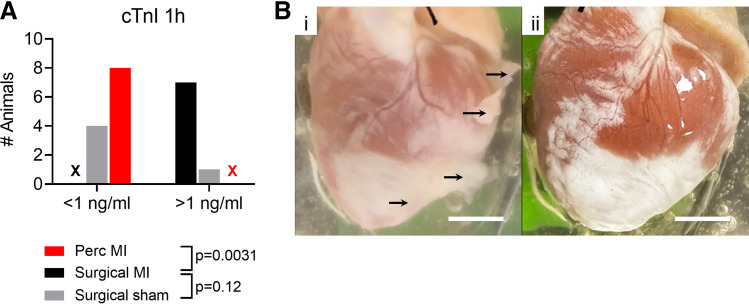
Improvements of the percutaneous myocardial infarction (MI) model compared with the surgical MI model. *A*: cardiac troponin I (cTnI) levels 1-h postoperative. Two-sided Fisher’s exact test resulted in percutaneous MI vs. surgical MI: *P* = 0.0031; surgical sham vs. surgical MI: *P* = 0.12. Cross indicates absence of values for that category. *B*: photographs of example hearts at 8-wk postocclusion, subjected to either the surgical MI procedure (i) or the percutaneous MI procedure (*ii*). Scale bars indicate 1 cm. Black arrows indicate adhesions.

The surgical MI procedure involves a thoracotomy, disruption to pericardial structures, and an apical suture to manipulate the heart. All of these may introduce pathophysiological changes that would not be anticipated following myocardial infarction. One-hour troponin levels were measured, and the results are shown in Fig. 11*A.* One-hour cTnI was more frequently elevated >1 ng/mL in surgical versus percutaneous MI (*P* = 0.0031) which may reflect myocardial damage that is unrelated to the coronary occlusion. Furthermore, after 8 wk, epicardial adhesions could be seen in surgical MI hearts, but not in percutaneous MI hearts (Fig. 11*B*).

## DISCUSSION

Here, we provide a detailed description of a novel approach for percutaneous induction of MI in rabbits weighing between 2.5 and 3.5 kg by occlusion of a coronary artery using a microcatheter tip. Our results indicate that the myocardial injury resulting from this novel method is equivalent to surgical ligation in terms of ECG changes, 24-h cTnI, LVEF at 8 wk, and histological appearance. Importantly, unlike the surgical ligation model, our technique avoids damage to the pericardium, eliminating epicardial adhesions. Moreover, this approach represents a refinement of the existing surgical procedure, aligning with the principles of the 3Rs (reduction, refinement, and replacement) in animal research (35).

In this study, percutaneous and surgical MI procedures resulted in comparable average EF and scar size with a similar spread of data points. Power calculations indicate that the group sizes used in this study could resolve minimum relative differences in EF of greater than 25% and greater than 40% for scar size. Similar power calculations based on the estimates of the variance of these two biomarkers indicate that the reported group sizes allowed minimum differences in variances of 50% to be resolved. A considerably larger group size would be required to resolve smaller differences of means and variances that may still be considered functionally significant (e.g., 10–15%), thus the current study is limited in the extent it can determine the equivalence of the two procedures on the rabbit heart.

### Catheter Choice for Percutaneous Procedures

Using ex vivo MRI, we determined the range of vessel diameters in rabbits weighing 2.5–3.5 kg, specifically the diameters of the ascending aorta, aortic root, left coronary ostium, and the left coronary artery proximal to the occlusion location. Given an average diameter at the occlusion site of 0.63 mm, we propose that microcatheters with a diameter larger than 1.9 Fr are not appropriate for smaller-sized rabbits. The use of larger microcatheters may increase the risk of left main coronary artery occlusion, perforation, or vasospasm. In contrast, smaller catheters (≤1.5 Fr) would enable the operator to reliably target distal occlusion sites, including access to branched coronary arteries.

### Coronary Occlusion Using a Microcatheter Tip

To our knowledge, the simple technique of using a catheter tip to occlude the coronary artery has not been reported. The external diameter of the microcatheter tips used in this study was smaller than the lumen of the target vessel (by ∼100–200 μm on average). Fluoroscopy 1 h after tip deployment confirmed that the position remained unchanged. Subsequent histology and MRI at 6–8 wk postocclusion demonstrated that the percutaneous MI procedure resulted in a transmural infarct comparable with that of a thoracotomy procedure as shown in Figs. 9 and 10. Taken together, these data suggest that the introduction of a catheter tip is a suitable method for coronary occlusion.

### Mortality Rates

Postoperative mortality rates were not significantly different from the thoracotomy procedure and were comparable with mortality rates published by others (23, 25). The single postoperative death in this study occurred 1 day before the planned euthanasia at 8 wk. This rabbit had a 24-h cTnI level of >180 ng/mL and an EF of 35%, which are both indicative of a large MI.

### Use of Single Fluoroscopic Projection

Variation in the occlusion site, combined with variable coronary anatomy in the rabbit, results in differences in the size and characteristics of the resulting MI (22, 36). Using a portable C-arm with a single anterior-posterior fluoroscopic projection, we found that tip deployment at the same position on fluoroscopy can result in a range of scar characteristics (Fig. 9). This suggests that there are important aspects of the vascular anatomy that are difficult to visualize using a single projection. Although the use of multiple projections could improve the accuracy of tip deployment, they are likely to lengthen the procedure, require more contrast agents, and consequently increase the risk of complications. In our experience, it is crucial to minimize the duration that the angiographic catheter remains coaxial to the coronary ostium so that the coronary blood flow is not obstructed from the level of the ostium. It is also important to minimize the total procedure length as rabbits are sensitive to any procedure under anesthesia (37). Given the variability in rabbit coronary vasculature, the apical artery was targeted for occlusion, as it is relatively consistent and is clearly visible using a single projection. Therefore, we propose that a single projection is generally adequate to provide sufficient visualization to guide the catheter to a level that will cause a significant infarct of variable size. This variability can be advantageous in experimental studies by generating a range of biomarker values whose interrelationships can be studied through correlation.

### The 3Rs of Animal Welfare

The design of animal research is driven by the 3Rs of animal welfare (reduction, refinement, and replacement) (35). The percutaneous procedure is a refinement over the surgical ligation procedure as it does not require a thoracotomy. The number of animals used in this study was reduced by using some data from parallel studies to provide an accurate means and SD of the surgical ligation procedure, hence the higher numbers in this group.

### Alternative Locations for Vascular Access

Katsanos et al. have reported a successful percutaneous MI procedure using transauricular vascular access in combination with the Seldinger approach. They also used a 4-Fr angiographic catheter in similar-sized rabbits (2.5–3.5 kg) (25). Two main advantages of their technique are *1*) avoiding a surgical wound in the neck and *2*) avoiding disruption to the carotid circulation. Alternatively, the femoral artery can be used for vascular access (38, 39). However, this approach involves guiding the catheters around the aortic arch, which may mechanically stress the vessel and therefore risk damage. In our study, no wound infections or neurological complications were seen; nor were these reported by others (22, 23). This suggests that the carotid artery is an acceptable location for a percutaneous procedure.

### Differences in Sex and Age

Because of restrictions in breeding arrangements by the supplier and latterly housing restrictions in the animal facility following COVID-19, only males could be used in this study. Future studies on the pathophysiology of MI should include females to reveal potential sex differences. There has been a large study in female rabbits using a closed-chest model, where they investigated the coronary anatomy and concluded that there was no difference between male and female rabbits (22). Similarly, aged rabbits show differences in scar remodeling (40), as well as hemodynamic and structural changes (41) compared with young rabbits, so researchers should consider the age-appropriate cohort of rabbits for their studies. However, in general, arteries slowly dilate with age (42), indicating that the catheter tip would also fit in the apical vessels of older rabbits. We therefore anticipate that this protocol would be applicable to both female and older rabbits of similar size.

### Perspectives and Significance

The induction of MI by a percutaneous route is a refinement of the traditional thoracotomy procedure in terms of procedural morbidity, as well as removing epicardial adhesions, which is beneficial for experimental studies. Studies of the regeneration and repair of the myocardial scar with artificial heart tissue would benefit from a percutaneous procedure because the surface tissue will more closely resemble the in vivo pathological substrate. Typically, experimental cellular constructs are implanted during the same thoracotomy procedure as the coronary artery occlusion, i.e., directly after the ischemic event (43–45). Serial thoracotomies several weeks apart allowing implanting of constructs in mature scars is avoided because of ethical and surgical complications (46). Using the percutaneous procedure to induce ischemia, grafting could be performed through a thoracotomy procedure many weeks later, onto a normal epicardial surface that is absent of adhesions thereby allowing study of graft-host coupling (47).

### Conclusions

In summary, percutaneous coronary occlusion by microcatheter tip deployment is a feasible approach in rabbits (2.5–3.5 kg) and produces an MI with similar characteristics to surgical ligation, with lower procedural trauma and without epicardial adhesions.

## SUPPLEMENTAL DATA

10.17504/protocols.io.yxmvm3b39l3p/v1 Supplemental Video File: dx.doi.org/10.17504/protocols.io.yxmvm3b39l3p/v1.

## GRANTS

This study was funded by British Heart Foundation (BHF) Centre for Regenerative Medicine Grant CRMR/21/290009 (to E.H., C.D., R.M., and G.S.), BHF Centre of Research Excellence Grant Number RE/18/63/42/17 (to M.F., E.H., R.M., and G.S.), BHF Project Grant PG/19/55/34545 (to E.B., R.M., M.D., and G.S.), BHF Project Grant PG/21/10545 (to C.D.), and Physiological Society Conference Attendance Award Grant 41317-FR (to E.H.).

## DISCLAIMERS

The content is solely the authors’ responsibility and does not necessarily represent the official views of the School of Cardiovascular and Metabolic Health or that of the University of Glasgow.

## DISCLOSURES

No conflicts of interest, financial or otherwise, are declared by the authors.

## AUTHOR CONTRIBUTIONS

M.F., E.H., C.D., R.M., and G.S. conceived and designed research; M.F., E.H., E.B., and M.D. performed experiments; M.F., E.H., and E.B. analyzed data; M.F., E.H., E.B., F.B., R.M., and G.S. interpreted results of experiments; M.F., E.H., and E.B. prepared figures; M.F. and E.H. drafted manuscript; M.F., E.H., E.B., F.B., R.M., and G.S. edited and revised manuscript; M.F., E.H., E.B., M.D., F.B., C.D., R.M., and G.S. approved final version of manuscript.
